# "I should live and finish it": A qualitative inquiry into Turkish women's menopause experience

**DOI:** 10.1186/1471-2296-10-2

**Published:** 2009-01-09

**Authors:** Serap Y Cifcili, Mehmet Akman, Abdullah Demirkol, Pemra C Unalan, Etienne Vermeire

**Affiliations:** 1 Marmara Medical School, Family Medicine Department, Tophanelioğlu c. No: 15-17 34662 Altunizade, Istanbul, Turkey; 2 Royal Prince Alfred Hospital, Drug Health Services, and University Of Sydney, Sydney NSW, Australia; 3 University of Antwerp, Department of Primary Care, Interdisciplinary Care and Geriatrics, Department of Nursing and Midwifery, Faculty of Medicine, Antwerp, Belgium

## Abstract

**Background:**

While bio-medically, menopause could be treated as an illness, from a psychosocial and cultural perspective it could be seen as a "natural" process without requiring medication unless severe symptoms are present.

Our objective is to explore the perceptions of Turkish women regarding menopause and Hormone Therapy (HT) to provide health care workers with an insight into the needs and expectations of postmenopausal women.

**Methods:**

A qualitative inquiry through semi-structured, in-depth interviews was used to explore the study questions. We used a purposive sampling and included an equal number of participants who complained about the climacteric symptoms and those who visited the outpatient department for a problem other than climacteric symptoms but when asked declared that they had been experiencing climacteric symptoms. The interview questions focused on two areas; 1) knowledge, experiences, attitudes and beliefs about menopause and; 2) menopause-related experiences and ways to cope with menopause and perception of HT.

**Results:**

Most of the participants defined menopause as a natural transition process that one should go through. Cleanliness, maturity, comfort of not having a period and positive changes in health behaviour were the concepts positively attributed to menopause, whereas hot flushes, getting old and difficulties in relationships were the negatives. Osteoporosis was an important concern for most of the participants. To deal with the symptoms, the non-pharmacological options were mostly favoured.

**Conclusion:**

To our knowledge, this is the first qualitative study which focuses on Turkish women's menopausal experiences. Menopause was thought to be a natural process which was characterised by positive and negative features. Understanding these features and their implications in these women's lives may assist healthcare workers in helping their clients with menopause.

## Background

While bio-medically, menopause could be treated as an illness or a symptomatic process, or even a hormone deficiency for which some sort of treatment can be utilized, from a more psychosocial and cultural perspective it could be seen as a phase in a woman's life, not requiring medication unless severe symptoms are present [1].

The influence of different opinions about menopause has been discussed by researchers. It is asserted that, encouraged by the medical industry, menopause was "medicalized", and this medical paradigm of menopause was associated with negative attitudes and consequently a negative menopausal experience [2-4].

In this context, from the early 1980s until the mid 1990s, Hormone Therapy (HT) was seen as the answer to all menopause-related problems, as well as the prevention of cardiovascular events [5]. This concept has since been revised following the results of two large randomized trials: the Heart and Estrogen/progestin Replacement Study (HERS) in 1998 [6] and the Women's Health Initiative (WHI) in 2002 [7]. The current consensus on the utilization of HT has been limited to symptom relief and maintenance of quality of life [8-10].

Although not population-based, the data collected from the outpatient setting of a gynaecological clinic in Turkey suggests that the frequency of any symptom related to menopause experienced by women was 82.3% [11]. Two studies from Primary Health Care Centres in Turkey report the frequency of vasomotor symptoms as approximately 46% [12,13]. In these studies, although symptom reporting was high, HT use was lower than that of western societies [12,13].

The feminist paradigm has viewed menopause as a life transition and problems of this time as normal processes, and not indicators of a "disease" [4]. In contrast to a biological understanding of menopause, feminists argued that menopause drew its meaning from societal attitudes towards ageing, the role of women in the social structure and the prevalent view of women as reproductive beings [14].

As Turkey geographically lies between east and west, and the social life-style also shows a broad variety, some women live more traditionally while some others, relatively small in number, live more a "western" style supported by the laws of the secular republic [15]. Regardless of how they live, currently most women in Turkey are exposed to the western concepts of health and well-being through media and the medical establishment which follows the European tradition.

This study aims to explore the perceptions of Turkish women about menopause and HT in order to provide health care workers with insights into the needs and expectations of menopausal women.

## Methods

This study used semi-structured interviews to explore experiences, beliefs and attitudes of Turkish women about menopause and HT. The questions which were used in these semi-structured interviews are presented in Table 1. As this involved collecting sensitive, personal information regarding the participants' experiences, knowledge and attitude towards menopause and HT, a qualitative approach was used.

**Table 1:** Outline of the semi structured interview

1. What does the term "menopause" mean to you?
2. What are your feelings about menopause?

3. Is there any change in your life related with menopause? OR Does it affect your life in any way?

4. Is there any relation between menopause and general health? OR Does menopause affect your general health?

5. Did you ever talk to anyone about your menopausal complaints? OR Did you get any help/support from anyone?

6. How do you deal with menopausal symptoms?

7- If not mentioned before: Of what does the term "hormone" remind you?
Did your doctor suggest that you use hormones? IF NOT, what would you feel if he/she did so?

The study was approved by the Ethical Committee of Marmara Medical School.

Sixteen women who responded to the study invitation were recruited over a three month period from the women who presented themselves to the Family Medicine and Menopause Outpatient Clinics of a University Hospital in Turkey.

It is important to note that, in Turkey, primary care does not have a "gate-keeper role" yet. In large cities like Istanbul, people can visit any hospital without a referral, including university hospitals. Family Medicine Clinics serve as a first port of call for service in these hospital settings.

The inclusion criteria were having experienced amenorrhea for at least 6 months and a minimum of one vasomotor symptom, as well as agreement to participate in the study. To develop a comprehensive understanding of the issue, we used a purposive sampling and included an equal number of participants who had experienced climacteric symptoms at the time and complained about the symptom (had sought help) as well as those who visited the outpatient department for a problem other than climacteric symptoms but when asked declared that they had been experiencing climacteric symptoms (had not sought help).

Once an individual agreed to participate in the study, she was interviewed by one of the two interviewers, who did not have a direct doctor-patient relationship with the participants. Interviews took place in one of the two offices of the Department of Family Medicine. Both offices were similar in size and internal layout. Interviewee and interviewer sat on similar office chairs, at the same level. Ten of these participants were interviewed on the day of their presentation to the clinics once they had been seen by their doctors. The other six who could not stay on but agreed to participate were interviewed on a later date that was convenient for them. During this process, five women who originally agreed to be interviewed later declined to participate in the study because of time constraints.

The interview questions focused on two areas:

a) knowledge, experiences, attitudes and beliefs about menopause and menopause-related experiences,

b) ways of coping with menopause and perception of HT.

The interviews were audio taped. After each interview, the interviewers made notes on what they observed and then the tapes were transcribed within 48 hours of the interviews. On four occasions after the transcription, we contacted the participants again in order to clarify the meaning of their statements.

Each transcript was then distributed to the other researchers in order to conduct a thematic analysis by using Strauss' Constant Comparison Method. The process is summarised below:

• SC, MA and AD separately read all the notes made by the interviewers as well as the transcripts.

• They looked for indicators of categories in the texts and then named them and coded these categories on the document.

• They compared the notes for differences and consistencies.

• The consistencies between the codes revealed the categories.

• All categories were then fed back into the subsequent interviews as probing questions, if they did not arise during the interview spontaneously, to explore the extent to which these experiences were shared among the participants.

• Each category was considered saturated when no new codes related to it were formed.

• Once all three investigators agreed that the categories were saturated the recruitment process was finalised.

• At the end of this, certain categories became more centrally focused and these are presented in this paper's result section as emerging themes.

## Results

The participants were aged 42–53. Two of them had very little (1–3 years) education, three had 5–8 years, and eight had 11 years of education, while two had a university degree. Most of the participants were from the middle class, two thirds of them were employed or retired and thus had a personal income. The majority (twelve) were married, two were widowed, and two were divorced. Two were living alone.

Eight of the participants visited the Gynaecology Clinic of Marmara Medical School and eight of them were recruited from the Family Medicine Outpatient Clinic. The most common symptom was hot flushes, whereas irritability, loss of sexual interest, insomnia, muscle pain and forgetfulness were other mentioned symptoms.

The data showed menopause to be both a positive as well as a negative experience and indicated ways they coped with menopause. The quotes used in the text were chosen according to the themes that emerged from the text. Numbers in parenthesis following quotes indicate the age of the participant.

### Menopause as a positive experience

Some participants associated menopause with "cleanliness" because of not having a period. When talking about cleanliness, participants said that before menopause they were concerned that those around them might notice they had been menstruating. They also said that before menopause they had vaginal discharge more often.

Menopause was also perceived as "comforting" as it removed the risk for pregnancy as well as the termination of menstrual symptoms. One participant was pleased that her menstrual migraine had disappeared along with the menstruation and, according to her; none of the symptoms of menopause were comparable to that of migraine.

"Cleanliness, not having menstruation every month, when I go out I don't have to check out if my clothes are stained or not." (51)

"I don't have unpleasant discharges and those menstrual cramps. That is a good thing." (50)

"I became more relaxed, as if I was being stressed about menses. At least I don't have to be concerned about pregnancy. (52)

#### 1- Menopause: change in health behaviour

The participants in both groups were aware of a number of health problems related to menopause and indicated that they were following their routine health controls, such as mammography, osteodensitometry, pap-smear testing etcetera, much better than they had prior to menopause. They also shared their experiences with their peers.

One of the participants clearly defined menopause as "the beginning of a new life" in which she is more conscious and more concerned about her health.

"After menopause I started to take care of myself much better. I do my smear check-up, mammography and so on. I meet with my friends once in two weeks. That's all we talk about. We ask each other about mammography, etc. Kind of menopause get-togethers..." (53)

Some participants mentioned their mothers and how they had had diseases which they linked to menopause and that they had not been able to access proper health care because of lack of knowledge.

#### 2- Maturity

Menopause also was defined as maturity, and the participants stated that as they went through "all stages of femininity" they could guide youngsters about these issues and therefore they felt more mature.

"I become more experienced every day, I am more optimistic than before, I review my whole life and I can guide my children and other youngsters around me."(51)

Some participants stated that they were more concerned about menopause and it was not as bad as they had thought it would be.

"Before menopause I thought that women feel "old" after menopause, that the femininity ended, an old woman with grandchildren etc. After menopause however, I understand that I was wrong. Now I think that menopause is natural and supposed to happen. These hot flushes sometimes make me sick, other than that it is natural". (52)

### Menopause as a negative experience

Although the participants attributed some positive meanings to menopause, such as cleanliness or comfort, they also experienced difficulties during this time.

While some participants spoke of menopause as comforting because of not having a period, some others in contrast defined menstruation as comforting. Since they were not menstruating any more, they could not experience that relieving effect of menstruating.

"Once the period begins, a woman becomes light as a bird, one who is menstruating is youthful, one who does not has aches, becomes frustrated."(52)

#### 1- Hot flushes

All participants experienced hot flushes to some degree which seemed to be the most disturbing problem. Participants of the two groups did not differ in terms of vasomotor symptom severity.

For some, hot flushes were a social as well as a physical problem since the people around them might notice them. Severe hot flushes were perceived to be interfering with the quality of life.

"These hot flushes, they get on my nerves. It can be easily seen by other people. Suddenly you become all red. I don't like this at all. I'm just about to go out. I'm all dressed up. And suddenly I sweat profusely, and I look like a drowned rat. Then I have to change all my clothes." (55)

#### 2- Getting old

Some participants associated menopause with getting older, a process they also saw as linked to osteoporosis, its complications and possible loss of function. These concerns seemed to lead them to take better care of their health.

"Menopause scares me. It's like I will get old and die. My bones will melt down and then I will die.......... Not getting old but shrinking down....It changes from person to person. Some people shrink down earlier....." (52)

"I can't do many things easily that I used to do. I think that in the beginning the bones weaken. Once your bones are not strong any more, your mobility becomes limited. You should be more careful so that your bones won't get broken." (49)

Most of the participants, who had sought help, thought that they were still young, not old enough for menopause. It appeared that there was a shared concern about the age of onset for menopause, that is: the earlier the onset of menopause was, the more severe the menopausal symptoms would be.

#### 3- Difficulties in relationships

The participants mentioned family conflicts, especially problems with their partners. "Emotional instability or irritability" appeared to be the main reason for these conflicts. The women stated that they were very sensitive and irritable without any apparent reason and this irritability was placing their relationship with their families under stress.

"It is something very emotional, as if my brain commands me to fight with somebody. Actually there is no reason to fight." (53)

"I have lost my connection with my husband; I am irritated all the time......my irritability affected my relation with my children. I'd suddenly get angry and shout at them. Then I asked to myself why do I behave so?" (52)

The participants also spoke of their need for support at this difficult life stage and lack of family support seemed to worsen the problem. By support, the participants mostly talked about their expectations from family members to be more patient and tolerant of their irritability.

"My family was insensitive and inconsiderate. Although he is a teacher, my husband was insensitive too. That was distressing to me. For example they were not considerate when I was irritable. They could not adjust to this period. When I spoke about this they told me that everyone goes through this, it is not only you. (45)"

Participants spoke of loss of sexual interest as a difficulty and a handicap for their relationship with their husbands. According to one of the participants even though she had lost her interest in sex she tried to continue her sexual practice to maintain her relationship with her husband.

Two of the participants said that "they had not been very much interested in sex" even before menopause. Some of the participants explained their sexual dysfunction because of vaginal dryness. Some of the participants downplayed the loss of sexual interest, either for themselves or both themselves and their partners. Only one referred to loss of sexual interest as an unpleasant experience.

"We're living like brother and sister now but this is not a problem for us." (53)

"Yes I lost my sexual interest and I don't care. Just my husband's disappointment makes me stressed and I force myself...." (45)

### Ways to cope with menopause

Although the participants described a number of ways to deal with menopause, the idea that these symptoms were temporary seemed to be the most helpful.

"I keep telling to myself that this is a natural phase, I should live and finish it."(52)

Seeking medical help appeared to be a way by which they coped with vasomotor symptoms. Those who sought help presented similar symptoms to those who did not seek help for menopause-related problems. These participants thought they needed to be medically checked during perimenopause, since they felt that they were not old enough to go through menopause. On further questioning, some of the participants who did not seek help were also being medically checked in terms of routine screening.

#### 1- Natural is healthy

In dealing with menopausal symptoms women seemed to prefer the ways they called "natural".

By "natural", women meant non-pharmacological methods such as diet, exercise and an herbal product which helps with hot flushes. They found these methods useful. Exercise was the most favoured among all of these.

"I feel better after I start to do exercise. I told myself that I've found the solution. I got over my bone problems with the help of exercise; I don't feel pain anymore."(45)

#### 2- Hormone therapy

Only one of the participants who used HT for five years described HT as an effective treatment choice.

"I was so unhappy when my doctor stopped my hormone therapy after one year, because all the symptoms of menopause started again. I wished the Americans had announced the results of the hormone study a bit later, so that I could still be using hormones nowadays."(55)

The others did not spontaneously speak of HT and when specifically asked, their attitude was mostly negative. They were concerned about side effects and risks. The strongest emphasis was on the risk of breast cancer related to HT use.

"I heard that HT causes cancer; uterus and breast cancer. They trigger cancer, which is why I don't consider using HT; I'm against hormones because I believe that it will trigger cancer cells in my body. Also a friend of mine used hormones and then she gained weight and became hairy."(51)

The source of the knowledge was mainly the media and friends rather than health professionals. This may be behind such negative views about HT. Some of the participants were confused and not certain about the accuracy of the information they had.

"...as far as I've heard, you get hairy when you use hormone, actually I don't know much..." (50)

"... they make you gain weight, I don't know actually, this is what they say."(43)

"...the rumour has it that hormones destroy some part of the body while it is repairing another part. That is why I am against using medication." (45)

Even the participants who sought help for their symptoms were not keen to use HT and it appeared that some of the medical advice was as confusing as that from the media and friends.

This may be explained by the confusion among medical practitioners regarding HT after the latest publications, such as WHI.

"When I told my doctor that I have stopped taking hormone pills, 'You have done the right thing' he said, 'Hormones impair the balance of the body."(46)

## Discussion

This study aimed to explore the perceptions of Turkish women regarding menopause and HT, in order to provide health care workers with an insight into the needs and expectations of postmenopausal women.

The participants of this study were from a metropolis of 12 million, mostly from the middle socioeconomic level and relatively well-educated compared with the general female population of Turkey [16]. There were also a few less-educated participants, who had migrated to the city from small villages.

Before proceeding to the discussion, it is important to note that there is not a Turkish word to define menopause. Occasionally "adetten kesilme", which literally means "cessation of menses", is used to refer to menopause, which does not have any cultural sub-meaning. Most of the population uses the word menopause written down as "menopoz".

Following is the detailed discussion of these findings:

Most of the participants of our study referred to menopause as a 'natural transition period'. "Natural" was defined as an inevitable but difficult time that one should go through. According to the feminist paradigm menopause is viewed as a natural developmental process that signals a life transition [4]. In Biri et al.'s study, 75.4% of the study population have had no concerns about menopause and the authors suggested that this was because of the women's understanding of menopause as a natural phenomenon, as is often the case with women from east Asian societies [12]. In addition, they emphasized the fact that although nearly half of the population were experiencing vasomotor symptoms, they did not have any concerns about these symptoms. In our study, women who had menopause-related symptoms were purposively sampled. Regardless of their help-seeking behaviour, they thought that this time of their lives was "natural" and would "come to an end". Very similar expressions can also be found in a Western study [17].

A qualitative study from Sweden reports positive perceptions of menopause and discusses the fact that positive aspects of menopause might be belittled by the medical approach to it [3]. This might be due to asking only about negative aspects of menopause in most epidemiological studies reflecting the medical paradigm.

Maturity, the comfort of not experiencing menstrual symptoms and the freedom from the fear of pregnancy were emerging positive themes that were associated with menopause. These themes were consistent with literature [3,18]. Maturity was defined as "the wisdom of being older, being able to help youngsters and even the beginning of a new life". Similar positive meanings have been documented in some other studies from different countries [19,20].

In a study designed to explore Italian-Canadian women's views of menopause, the authors noted that menopause was perceived as a liberating period, especially in patriarchal societies [21].

Although Turkey can be considered a patriarchal society, none of the women specifically expressed a similar perception.

Menopause was defined as "cleanliness" by the majority of the participants. The "cleanliness" was about not having a period or vaginal leakage which they associated with menstruation. The same concept was referred to as "freedom" in the study mentioned above [21]. This association between not having periods and cleanliness may be a reflection of Islamic traditions, according to which women are not allowed to participate in any religious activity during the menstrual period. Many women both from rural and urban areas in Turkey occasionally use the term "I'm dirty" or "I got dirty" to refer to menstruation. After completing their period, women are obliged to "bathe" before starting religious practice again and this is traditionally called "cleaning". Hence if one does not menstruate, one is always "clean". In today's secular Turkey, many women do not practice all religious rituals, however they still traditionally use these terms.

While most of the participants defined menopause as a natural transition period, there were also some negative concepts linked to menopause which were expressed by women who sought help as well as those who did not. These negative features were: experiencing vasomotor symptoms, fear of getting old and loss of sexual interest.

Vasomotor symptoms; hot flushes and night sweats were the most emphasized and bothersome complaints. There is strong evidence from both longitudinal and cross-sectional observational studies that the menopausal transition causes vasomotor symptoms and these symptoms improve with oestrogen treatment [22]. In the USA after the production of inexpensive oestrogens, the biomedical paradigm was the primary paradigm within which menopause has been conceptualized. Menopause was described as a "clinical disorder of the ovary characterized by estrogen deficiency" [4]. In keeping with this view, it was suggested that all women should be given HT to treat their complaints. However, studies from eastern Asian countries showed much lower frequency of symptom reporting, and the idea that there is one universal menopausal syndrome is challenged [23]. A "biocultural" approach was proposed: the differences result from the inseparable interactions of biological and cultural/environmental influences on a woman's menopause experience [24].

In Turkey, cross-sectional studies have shown that vasomotor symptom frequency is similar to Western studies [12]. However, the participants who were experiencing severe symptoms also developed solutions to these symptoms such as exercise or self-reassurance.

Some participants said that they found vasomotor symptoms embarrassing because of their being noticed by people around them. Similar concerns were noted by Berterö [21].

Biri et al.'s cross-sectional study reports the frequency of psychosocial symptoms as between 41 and 64%, in which four different categories were defined as psychosocial complaints: anxiety, feeling of stress, touchiness and self-depression, and absent-mindedness [12]. In our study emotional instability and irritability were the most common psychosocial complaints. The irritable state was more defined as a temporary state of anger. Some of the participants described these experiences as "tides" i.e. as though they came in episodes. In different studies the prevalence of mood symptoms during menopause varies from 8% to 38% [22]. Many studies in Turkey have explored the relationship between depression and menopause, reflecting the common medical paradigm that menopause has severe negative effects on women, however more recent studies have denied this relationship [25]. In our study, the participants' irritable state seemed to be interfering with their family relationships and some of the participants expressed their feelings about the need for support and understanding from those around them. According to the findings of another study that explored depressive symptoms of Turkish women aged 40–65, the women who are being supported by their husbands had less depressive symptoms [25]. It appears that whether family support will help in reducing women's stress during this period is an important issue which needs further exploration.

Loss of sexual interest or vaginal dryness were other matters that women in this study expressed as problems that affect their relationship with their partners. This result is consistent with a cross-sectional survey covering 1805 postmenopausal women from six European countries, which concluded that women experience the menopause as a process that brings about mood and sexual changes which may impair their personal life [26]. In our study, although most of the participants talked about sexual dysfunction, they did not nominate it as a major problem. It can be said that cultural values and health beliefs influence perception of sexuality at the time of the menopause and will also influence the need for treatment [26].

While some of the participants welcomed the "maturity" and the status that came with menopause, they expressed negative feelings about "getting old" and problems associated with age, such as osteoporosis. Participants who sought help seemed to be more concerned about the age of onset of menopause rather than menopause itself. They felt that they were too young to go through menopause. This might be a reflection of the idea that midlife is a stage when individuals actively seek to remain young for as long as possible [19], where femininity is socially constructed as being youthful [20]. It is important to note that the participants did not focus on their body image, rather wanting to stay active and independent. They were scared that osteoporosis might weaken their bones and that in the end they would not be able to move independently. In a qualitative study, it was reported that only a few women linked osteoporosis to menopause, however in our study almost every participant referred to "bone problems", that is osteoporosis [3]. This may be due to ongoing media reports that put the emphasis on osteoporosis during menopause.

In our study, none of the participants spontaneously referred to HT as a treatment option. This is in keeping with the available data on HT usage in Turkey, which reports only 6.8% of women would use HT despite the high frequency of symptom reporting [11]. Some researchers suggested that women who perceive menopause as a natural phenomenon do not intend to use HT [27,28]. Before the publication of WHI, HT had been widely advocated as the answer for menopause-related problems [29,30]. It has often been suggested that this has resulted in medicalization of menopause, especially by western medicine [27,30,31]. When specifically asked about HT, women said they did not want to use HT because they thought it was unnatural and chemical. Another reason mentioned for unwillingness to use HT was negative perception towards medications in general. In another qualitative study, we explored the reasons for non-compliance with antihypertensive drugs; some participants had described drugs as "poison" [32]. This negative perception of medication together with regarding HT as "unnatural", may explain the reluctance of the participants to consider HT usage.

To deal with their symptoms many interviewees in this study tried life-style modification, which was partially helpful in handling their symptoms. Participants specifically emphasized that exercise was beneficial; a finding that is supported by another study [33]. This seems to be an opportunity to promote healthy lifestyle choices to this group of women not only for menopause-related symptoms but also for chronic conditions common at that age, such as diabetes and cardiovascular disease.

### Limitations and weaknesses of the study

This study does not seek to generalise its findings to the general population. It describes the experiences of the participants in depth in order to present their perceptions, strengths and expressed needs. It is possible that the findings of this study may be transferable to other settings in which middle class Turkish women from Muslim backgrounds reside.

The study sample reflects the demographics of the patient population attending the family medicine and gynaecology clinics of a university hospital, which are available to those who hold health insurance or can afford to pay the fee for service.

## Conclusion

To our knowledge, this is the first qualitative study which focuses on Turkish women's menopausal experiences. The perception of menopause was said to be a natural phase which was characterised by some positive and some negative features. Understanding these features and their implications in these women's lives may assist healthcare workers in helping their patients with menopause.

## Abbreviations

HT: Hormone Therapy; HERS: Heart and Estrogen/progestin Replacement Study; WHI: Women's Health Initiative; TMSO: The Turkish Society of Menopause and Osteoporosis.

## Competing interests

The authors declare that they have no competing interests.

## Authors' contributions

SYC participated in the design of the study, acquisition of data, analysis and interpretation of data and writing the manuscript. MA participated in the design of the study, analysis and interpretation of data and writing the manuscript. AD participated in the design of the study, analysis and interpretation of data and critically revised the manuscript. PCU participated in the design of the study and critically revised the manuscript. EV has critically revised the manuscript for important intellectual content. All authors read and approved the final manuscript.

## Pre-publication history

The pre-publication history for this paper can be accessed here:


